# Catamenial cyclic vomiting syndrome responding to oestrogen therapy: an adolescent case report

**DOI:** 10.11604/pamj.2019.33.286.17978

**Published:** 2019-08-06

**Authors:** Moulay El Mehdi El Hassani, Benali Saad, Moukit Mounir, Jaouad Kouach, Driss Moussaoui Rahali

**Affiliations:** 1 Faculté de Médicine et de Pharmacie de Fès, Fès, Maroc; 2 Service Gynécologie Obstétrique, Hôpital Militaire d’Instruction Mohamed V, Rabat, Maroc

**Keywords:** Cyclic vomiting syndrome, menstrual period, catamenial migraine, oestrogen

## Abstract

Cyclic vomiting syndrome (CVS) is defined by episodes of vomiting lasting from a few hours to several days, alternating with periods of no symptoms. Various symptoms can be associated with vomiting such as nausea, migraine or abdominal pain. Common triggers of CVS include infection, psychological stress and menstruation. CVS's diagnosis requires exclusion of alternative diseases particularly neurological and gastrointestinal. CVS shares many common features with catamenial migraine including treatment. We herein report a case of CVS in a 16 years old girl characterized by stereotypical vomiting attacks occurring in every menstrual period. Recurrent vomiting episodes began 2 years before admission. Given the negativity of paraclinical exams and the absence of response to different therapeutic approaches as well as the similarity with catamenial migraine, we treated our patient with permenstrual percutaneous oestrogen for six months. The evolution was marked by the disappearance of symptoms within the first month and the absence of their recurrence after treatment cessation during a follow-up of 6 years.

## Introduction

Cyclic vomiting syndrome (CVS) is a relatively rare disorder characterized by recurrent episodes of sudden severe nausea and vomiting interspaced with asymptomatic periods. CVS remains a clinical diagnosis based on three main criteria (ROME III consensus) [1, 2]. It was initially described in children but could occur in all age groups. Recent studies have demonstrated that it is increasingly recognised as a cause of nausea and vomiting in adults and is highly disabling [3, 4]. CVS is a heterogeneous syndrome whose pathogenesis is still unclear. Triggering factors of acute emetic episodes are similar to CVS's in both adults and children including infections (e.g. chronic sinusitis, upper respiratory infections), emotional and psychological stress, motion sickness, lack of sleep, physical exhaustion and certain food products (e.g. chocolate, cheese and monosodium glutamate) [5]. For some women, episodes are triggered by menstruation similarly to catamenial migraine. We herein report a case of an adolescent girl who had menstruation-related cyclic vomiting and for whom permenstrual percutaneous oestrogen therapy successfully prevented symptoms recurrence.

## Patient and observation

A 16 years old girl was addressed to our department for recurrent permenstrual vomiting. Her history began 2 years before admission with recurrent stereotypic episodes of vomiting separated by asymptomatic intervals. Her symptoms occurred with the onset of her menstrual period and lasted for 6 to 7 days. They resolved spontaneously after its end. Episodes became uncontrollable requiring repeated hospitalisations affecting her schooling. Her past medical and surgical history was without particularities. The age of menarche was 12; the duration of menstrual flow was 6 to7 days whereas its amount was medium. Menstrual cycle was regular and its mean duration was 28 days. During the physical examination, the patient's vital signs were stable, her weight was 45 kg for a height of 158 cm. She had a female morphology and the secondary sexual characteristics were normally developed. The clinical examination especially neurological and abdominal was normal. Laboratory blood tests: liver, renal and thyroid functions as well as rates of follicle stimulating hormone (FSH), luteinizing hormone (LH) and prolactin hormone (PRL) were normal. The urinary pregnancy and infections tests were negative. Pelvic and abdominal ultrasound was normal. Upper gastrointestinal endoscopy, abdominopelvic CT scans, EEG and brain MRI did not show any abnormalities. Management with symptomatic treatment (hydration, antiemetic and prokinetic drugs, proton pump inhibitors and intravenous Lorazepam) did not affect the duration nor the severity of symptom episodes. Conventional treatments such as tricyclic antidepressants and low dose oestrogen progesterone pills did not prevent the symptoms recurrence on the next menstrual period. Given this disabling permenstrual cyclic vomiting for which no explanation was procured by diverse paraclinical examinations nor was any response obtained by the different therapeutic approaches; cyclic vomiting syndrome was evoked. We thereby adopted a therapeutic approach similar to that of catamenial migraine. In so doing, we treated our patient with percutaneous oestrogen at the rate of one dose per day, starting 48 hours before the onset of menstrual period and lasting 6 to 7 days, for the total duration of six months. The evolution was spectacular with the occurrence of only one episode of vomiting in the first month and the disappearance of all symptoms in the following months, persisting even after treatment cessation. The follow-up time is 6 years.

## Discussion

CVS is a rare disorder diagnosed primarily in children and frequently misdiagnosed as acute gastroenteritis, gastroesophageal reflux disease, pancreatitis or as an eating disorder [6, 7]. Patients with CVS typically display recurrent stereotypic episodes of nausea and vomiting lasting hours or days, interspaced by asymptomatic intervals lasting weeks or months. Vomiting typically begins during the night or the early morning and peaks in the first hour occurring up to 13 times per hour [2]. Classically perceived as a pediatric disorder, CVS is being recognized increasingly in adults and adolescents. CV episodes in children cease spontaneously before puberty in most cases, but persistence to adult life is well acknowledged. CVS in adults can occur at any age, with an average onset age of 21 to 35 in limited case series [8]. Although the clinical features in each age group are similar [9]; some differences in symptoms profile, pathogenesis and associated comorbidities have been observed between adults and children [1]. The physiopathology of CVS has not been established yet, nonetheless recent investigations suggest that several factors, including metabolic disorders, migraine, anatomic dysregulation, hypothalamic-pituitary-adrenal axis defects, tendency for anxiety and chronic cannabis use could precipitate CVS in some cases. 68 to 80% of CVS attacks have associated trigger mechanisms. These include infection, psychological stress, physical stress, inadequate sleep, diet, motion sickness and onset of menses [5]. The catamenial migraine is linked to the fall of oestrogen during a physiological cycle or when stopping a pill in women on oral contraception [10]. By analogy to catamenial migraine, the catamenial character of CVS can be due to intolerance to the fall of oestrogen at the end of the menstrual cycle in the women and the teenage girls (as in our case). Up to 13% of postmenarchial girls develop CVS at the onset of the menstrual period (hence named catamenial CVS) [11]. It is probable that anticipatory anxiety and stress at the approach of menstruation jointly contribute to the triggering factors of vomiting attacks [8]. CVS remains a diagnosis based upon history and exclusion of alternative diagnoses. Rome III criteria include: stereotypical episodes of vomiting regarding acute onset and duration (less than 1 week), three or more discrete vomiting episodes in the prior year, absence of nausea and vomiting between episodes [12]. Supportive criteria include history or family history of migraine headaches [8]. The recurrent vomiting attacks interspaced with the symptoms-free periods of our case met the consensus criteria of CVS. Differential diagnosis of CVS is very broad and it is important to exclude other gastrointestinal disorders as well as extra-intestinal disorders, including metabolic and central nervous system diseases, renal disorders and psychogenic vomiting. The diagnostic approach to exclude these disorders in cases of recurrent vomiting includes: biochemical testing for electrolyte abnormalities; thyroid, liver and renal functions as well as hormonal and infections blood tests, upper gastrointestinal endoscopy, abdominal CT, encephalogram and brain MRI. In our case all these exams were practiced and normal. No specific therapy has been proven efficient for CVS in controlled trials. However, several empiric treatments have been effective [13]. Life style changes, prophylactic therapy and supportive care to ameliorate acute episodes are efficient treatments.

During the emetic phase, treatment consists of hydration; antiemetic, antianxiety and/or analgesic medications. Supportive care with fluid replacement and electrolyte control is often necessary. A constant intravenous infusion of 5-HT3 antagonist, Ondansetron, has shown an efficacy of about 60% if given early in the vomiting attack [14]. Lorazepam provides sedation that may lessen intractable nausea in some cases thus its addition if nausea persists [15]. Nevertheless, treatment with Lorazepam and other symptomatic medications did not prevent the symptoms recurrence. Some studies have investigated the drug therapy in preventing episodes of CVS and only a small number of maintenance therapies have so far been reported to be effective. Low dose tricyclic antidepressants, Sumatriptan and beta-blockers have been found to be effective in prevention of episodes [16]. Furthermore, if present, psychiatric disorders should be treated. If menstrual cycles trigger an attack, GnRHa injections or continuous birth control to block menses should be considered. Although in one hand, low-dose oestrogen oral contraceptives could help to prevent the vomiting attack in patients with menstruations-related CVS [15], oral contraceptives can also exacerbate symptoms on the other hand [13]. Administration of GnRHa down-regulates the pituitary-ovarian gonadal axis and reduces levels of both the gonadotropin luteinizing and follicle-stimulating hormones [17]. GnRHa suppresses ovulation and is found to have benefits in the treatment of premenstrual syndrome. However, the maximum treatment period is 6 months and hormone replacement therapy (oestrogen) has to be given to prevent side effects [18]. The fall of oestrogen at the end of the cycle being the triggering factor, the permenstrual oestrogen therapy in catamenial migraine is a logical therapeutic approach, yet its level of evidence remains quite low [14, 19]. The efficacy in this indication has nonetheless been demonstrated in 3 double-blind controlled studies with a dose of 1.5 mg per day started 48 hours before the menstruation period for a duration of 7 days. Benoteau [10] has successfully treated catamenial migraine with percutaneous oestrogen E2 (transdermal or patch) started 48 hours before menstruation for a duration of 6 to 7 days. Given the principal of similarity with catamenial migraine, it only makes sense to consider oestrogen therapy for premenstrual prophylaxis in catamenial CVS. Thus, because of our patient's young age as well as the side effects of GnRHa, we opted for a permenstrual transcutaneous oestrogen therapy as a prophylactic method given its efficacy in catamenial migraine and a relatively short period of cures. Our patient had transdermal oestrogen at the rate of 1 dose per day starting 48 hours before the onset of menstruation and continuing for 6 days. The results were spectacular with total cessation of vomiting from the first month of the treatment which was terminated after 6 months with no recurrence for the following 6 years.

## Conclusion

In conclusion, careful understanding of history, symptoms pattern and triggering factors should be obtained in patients with recurrent vomiting attacks. For women and especially teenage girls with menstruation-related CVS, we suggest that oestrogen therapy is an effective therapeutic method for preventing recurrence of vomiting episodes.

## Competing interests

The authors declare no competing interests.

## Authors’ contributions

All the authors have read and agreed to the final manuscript.
